# Use of Virtual Reality in the Education of Orthopaedic Procedures: A Randomised Control Study in Early Validation of a Novel Virtual Reality Simulator

**DOI:** 10.7759/cureus.45943

**Published:** 2023-09-25

**Authors:** Austin R Gomindes, Elizabeth S Adeeko, Chetan Khatri, Imran Ahmed, Simran Sehdev, William John Carlos, Thomas Ward, James Leverington, Luke Debenham, Andrew Metcalfe, Jayne Ward

**Affiliations:** 1 School of Medical and Dental Sciences, University of Birmingham, Birmingham, GBR; 2 Trauma and Orthopaedics, University Hospitals Coventry and Warwickshire, Coventry, GBR; 3 Trauma and Orthopaedics, University of Warwick, Warwick, GBR

**Keywords:** training effect, haptics, orthopaedics & traumatology, virtual reality in medical education, virtual reality simulation, virtual augmented reality, tfn-advanced proximal femoral nailing system (tfna), skills and simulation training

## Abstract

Background

Virtual reality (VR) simulation is a potential solution to the barriers surgical trainees are facing. There needs to be validation for its implementation within current training. We aimed to compare VR simulation to traditional methods in acquiring surgical skills for a TFN-ADVANCED™ Proximal Femoral Nailing System (TFNA; DePuy Synthes, Auckland, New Zealand) femoral nailing system.

Methods

Thirty-one surgical trainees were randomised to two groups: traditional-training group (control group) and a VR-training group (intervention group) for insertion of a short cephalomedullary TFNA nail. Both groups then inserted the same TFNA system into saw-bone femurs. Surveys evaluated validity of the relevant activities, perception of simulation, confidence, stress and anxiety. The primary outcomes were tip-apex distance (TAD) and user anxiety/confidence levels. Secondary outcomes included number of screw- and nail-guidewire insertion attempts, the time taken to complete and user validity of the VR system.

Results

There was no statistical difference in TAD between the intervention and control groups (9mm vs 15mm, p=0.0734). The only TAD at risk of cut-out was in the control group (25mm). There was no statistical difference in time taken (2547.5ss vs 2395ss, p=0.668), nail guide-wire attempts (two for both groups, p=0.355) and screw guide-wire attempts (one for both groups, p=0.702).

The control group versus intervention had higher anxiety levels (50% vs 33%) and had lower confidence (61% vs 84%).

Interpretation

There was no objective difference in performance on a saw-bone model between groups. However, this VR simulator resulted in more confidence and lower anxiety levels whilst performing a simulated TFNA. Whilst further studies with larger sample sizes and exploration of transfer validity to the operating theatre are required, this study does indicate potential benefits of VR within surgical training.

## Introduction

Surgical training faces multiple challenges in the modern age [1], which must be counteracted to allow for the continuous production of proficient surgeons. For example, the European Working Time Directive (EWTD) has restricted working hours, thus limiting theatre exposure and case numbers for trainees [2,3]. These hurdles further decrease junior trainee experiences and consequently their necessary logbook numbers [4]. As such there need to be provisions to ensure accessible and safe opportunities to allow for adequate training in a shorter available time.

Virtual reality (VR) is the creation of a 3-D virtual environment via computer software which is experienced by the user as if it is real [5]. VR software and simulation have been considered as a possible method of formal training within surgical training in recent years [6-10]. Surgeons must maintain the ethical principle of non-maleficence and the General Medical Council (GMC) highlights the importance of actions being in the best interests of our patients, so it is clear patients should not be seen as just test subjects [11]. However, it is paramount for trainees to practice surgical techniques as accurately as possible.

It is this conundrum where VR training is a potential solution and has resultantly been adopted within the training curriculum in locations such as Denmark [12]. Whilst there is currently limited information regarding the transferability of the skills acquired from virtual reality to the real world [7] there is evidence of assistance with acquisition of relevant psychomotor skills, such as precise handling of instruments [7,12], objective technical competence [5,13] and familiarisation of real surgical procedures at least to a basic level [10,14]. VR simulation has also been shown to be a safe training tool, with a lower complication rate and no statistical difference in safety metrics when compared to the cadaveric gold standard in shoulder arthroscopy [15]. This was also suggested in Sugand et al.’s work with dynamic hip screw (DHS) simulation and much-reduced rates of cut-out risk [7]. Not only this, but virtual reality has also allowed for insight into factors with much wider implications than just the surgical procedure at hand such as potential career development [10] and a user’s accurate self-perception of skills [16].

Another important consideration from The Royal College of Surgeons is the impact of surgical performance anxiety on both surgeons and potentially their patients’ outcomes [17,18]. Whilst the effects of human factors on surgical performance are well documented these anxieties are yet to be fully explored. Surgeons are becoming more aware of negative psychological effects such as poor confidence or burnout on practitioners and the potential impacts this may have on patients [19].

Additionally, though there is support for the role of VR in formal training by users [20,21], there is limited evidence on how best to introduce this as an element into the UK medical education system [22]. There have been advances made within feasibility, validity, and reliability of VR in other surgical fields, for example general surgery [23], endoscopy [24], laparoscopic procedures [25] and various others [26]. However, there remains limited evidence within the orthopaedic field.

Aims

This study aims to determine if there was a difference between VR and traditional methods (using Optech notes) of preparation (master-apprentice), in procedure competence and efficiency. Additionally, this study aims to compare the psychological effects of both training methods on users and establish the acceptability and validity of a VR simulator.

Outcomes

Primary Outcomes

Primary outcomes were 1) to assess whether VR may be beneficial in improving users’ confidence, stress and anxiety levels when used in preparation of surgical procedures and 2) to assess acceptability of VR training among a surgical trainee population.

Secondary Outcomes

Secondary outcomes were 1) to assess the effects of VR on surgical practical effectiveness (tip-apex distance (TAD), time taken, and guidewire insertion attempts) and 2) to assess face validity and content validity of a VR trauma simulator.

## Materials and methods

This work was completed as a randomised controlled study and is reported in accordance with the CONSORT 2010 guidelines [27].

Setting

TAD is the distance from the screw tip to the apex of the femoral head on anteroposterior (AP) and lateral views [12]. TAD should be below 25mm in order to prevent DHS cut-out or failure, which most often happens if the screw is placed too superior or too anterior. The study took place during a purpose-design teaching session at a large, tertiary hospital (University Hospitals Coventry & Warwickshire). Participants were provided with information prior to the event and gave informed consent. Participation in this study was voluntary and did not impede ability to facilitate participation or completion of the teaching session.

Participants

The inclusion criteria were undergraduate and junior surgical trainees (postgraduate years 1-4 [PGY1-4]).

The exclusion criteria were specialist registrar trainees and consultants (≥PGY5), study assessors and course trainers.

Design

Participants were separated into groups and were cluster-randomised via a non-sequential random-number generator. They were allocated to one of two groups: (i) VR-training group (intervention group) and (ii) traditional-training group (control group).

Intervention

This group conducted a VR simulation of inserting the same TFN-ADVANCED™ Proximal Femoral Nailing System (TFNA; DePuy Synthes, Auckland, New Zealand) system and similarly could discuss steps with a present trainer. The VR simulator for this study was the DePuy Synthes Oculus Rift S powered by PIXELMOLKEREI. This is a computer (PC) powered VR system repurposed from the gaming industry with multiple surgical procedure simulations in-built within it. The user interface comprised a halo headband with incorporated visual lenses and two handheld controllers that detected user movements. 

Control

Traditional training methods can comprise laboratory skills, reading of surgical text and supervisor verbal feedback (master-apprentice) [28]. The traditional training group training consisted of operative technique (Optech) notes for insertion of the TFNA as provided by the manufacturer [29] and the steps of the procedure were discussed verbally with a trainer.

Nine out of the 28 participants had previous experience with virtual reality simulation (all of which was surgical in nature) and three of those had experience with an Oculus system. The most common operative experience level was completion of a range between 10 to 50 operative hip procedures (19 out of 28). All the participants had some experience in either observing/assisting/performing hip surgery, three participants had >50. Twenty-two of the 31 participants were male, 24 were right-handed and the median age was 28 years old.

Both groups had 20 minutes to complete the training task and subsequently inserted the same TFNA system onto dry saw-bone model femurs. This was facilitated by trainers from whom participants could ask questions.

Outcome measures

**Figure 1:**
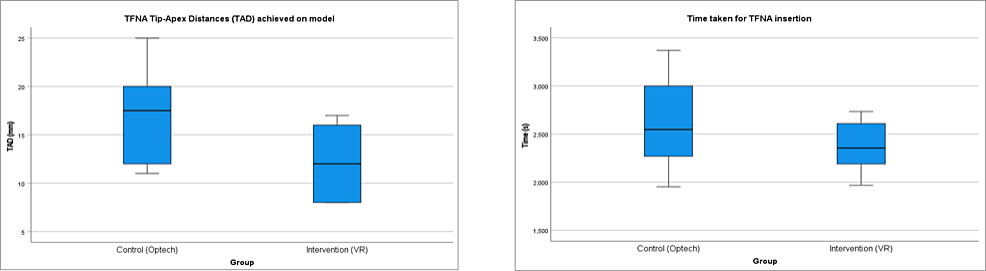
Depictions of quantitative data i) Comparison of median TAD (mm) between control and intervention groups during bone model practical. ii) Comparison of the number of attempts taken to insert TFNA nail guidewire (a and b) and TFNA screw guidewire (c and d) between control and intervention groups during bone model practical.

Quantitative practical measures were recorded during the bone model activity including the number of entry guidewire insertion attempts, number of lag-screw guidewire insertion attempts, time taken to complete procedure and the TAD (Figure 1). 

Likelihood of clinical failure is highly related to a complication known as “cut-out” of the screw which describes the expulsion of the lag-screw from the femoral head. This is related to the TAD [30] which represents distance of the tip of the screw to the apex of the femoral head [31]. It has been shown a TAD ≥25mm is related to high rates of failure [31,32].

Participants were also given surveys to complete at three time points during the process: 1. Before completing any study activity (Questionnaire 1 - Q1); 2. After completing the training activity i.e. traditional methods or VR (Questionnaire 2 - Q2); 3. After completing the bone-model insertion activity (Questionnaire 3 - Q3).

The assessment questionnaires were developed based on the work of Sugand et al.’s team [21] due to the ability to assess validity and acceptability in their research. Each of these surveys included 25 questions regarding validity of the relevant activity, which describes how well the intervention corresponds to its intended domain. We assessed content validity: if the VR system includes all the necessary components to achieve its aim, and face validity; how accurate the user’s subjective experience would be.

We assessed psychological measures including confidence, stress and perceptions of simulation and were assessed via 7-point Likert Scale. Participant anxiety levels during the study were assessed via the verified State-Trait Anxiety Inventory (STAI-6) score which enables a result that can categorise into anxiety levels (no/low anxiety, moderate anxiety, high anxiety) using both negative indicators (I was tensed, I was worried, I was upset) and positive indicators (I was content, I was calm, I was relaxed) [21,33-36].

Blinding

It was not possible to blind participants. Assessors remained blinded to the type of training (traditional or VR) when performing data collection and analysis. Trainers in the dry bone session were also blinded to the type of training received.

## Results

User perception

Acceptability of Simulation Prior to Participation

On starting the study, most participants agreed simulators should be compulsory within surgical training (85%) and felt it should be offered earlier in their training (88%). Also 100% of participants entered the study with a preference to prepare for surgical procedures with “hands-on” practice compared to reading information. Opinions were mixed on simulations' superiority to current traditional training methods with the majority being neutral (57%), only 21% agreeing and 18% disagreeing. On initial questioning, 79% of participants felt stressed about attempting to insert the TFNA and most participants (80%) were unable to state they felt confident in attempting this.

Face Validity

At the final time point, 100% of VR participants felt the simulator had an overall realistic appearance with a realistic view of the instruments. Ninety-two percent of participants also agreed that the operative movements were realistically exhibited in the simulation, with 42% strongly believing this. In addition, 75% of participants stated that when using the simulator, they felt as if they were performing the procedure in a real-life situation. Comparatively, only 40% of the control group participants felt the overall appearance of the traditional methods using Optech notes were realistic, but 80% felt the specific diagrammatic depiction of the surgical instruments was realistic. Just 20% of the control participants felt that the required movements were realistically exhibited and 60% of participants either moderately or strongly felt they did not have a realistic impression when preparing with the Optec notes.

Content Validity

When referring to the accuracy of information within the Optech notes, the control group at time point 2 (after completion of training) scored highly, with 53% stating the steps were accurate, 73% that the flow of steps was accurate and 73% that the anatomical components were accurate. One hundred percent of the group also felt the instructions were relevant to the procedure and 66% felt they had a better understanding after reading the notes. After completion of the dry bone model these scores largely increased with 66% agreeing with step accuracy, 80% stating the anatomy was accurate and 87% having a better understanding of the procedure. In this control group it was also seen at time point 2 that only 34% of participants felt prepared to attempt the procedure which increased to 46% at time point 3.

Comparatively, the intervention group had even more positive initial views regarding the simulator’s content with 100% of participants stating the steps were accurate, their flow was accurate and having a better understanding of the procedure after the simulation. Eighty-four percent of participants felt that the anatomy depicted was accurate and 100% of participants stated having a better understanding of the procedure. When reassessing at time point 3, 92% felt the steps and their flow were accurate, 100% felt the anatomy was accurate and 84% felt the instructions were relevant. One hundred percent still thought they had a better understanding and at both time points 62% of participants felt prepared to attempt the procedure.

Acceptability of Training Methods

When investigating participant perception of their experience with the training activity at time point 2, the intervention group stated that 92% enjoyed using the simulation and thought the simulator was easy to get used to, which increased to 100% at time point 3. At time point 2, 100% of the intervention group agreed or felt neutral to the idea that the simulator provided an unthreatening learning environment, would like to train further on the simulator, would want to practice on a simulator before performing the procedure on a patient and would recommend simulation to their colleagues. In comparison at the next time point, 100% of participants agreed with all the above elements.

Also, at the second time point, 100% of participants felt simulation should be offered earlier within surgical training as well as be compulsory within surgical training as an adjunct to traditional training methods. Sixty-two percent of the intervention participants stated they thought simulators were better than traditional training which increased to 69% at the final time point. The 100% acceptability regarding those elements continued at the third time point. Also, at time point 3, 77% of the intervention group stated they would feel safer being operated on by a surgeon who had trained with simulators in addition to traditional training methods, which differed from the 67% seen in the control group.

The control group again had differing perceptions elsewhere, as only 53% of participants thought reading notes was easy to get used to and enjoyed using them, and whilst enjoyment increased slightly to 58% at the next time point, ease of familiarity decreased to 47%. Seventy-three percent stated they would want to train further with the Optech notes and would recommend them to a colleague, but it was concerning that only 67% of users thought it established an unthreatening learning environment. On closer inspection, 13% thought it was moderately or strongly threatening and only 26% of participants would prefer using notes or reading to practical activities such as simulation across both time points. In a similar stance to the intervention group at the last time point, 93% thought simulation should be introduced earlier in training and 100% agreed or were neutral to the idea of simulation being compulsory. Across both time points 67% of participants would feel safer to be operated on by a surgeon who had trained with both traditional and simulated modalities.

Confidence/Stress/Anxiety

Analysis of participant confidence throughout the study showed that 69% of the intervention group felt at least mild confidence in attempting TFNA insertion at time point 2, which increased to 84% at time point 3. On further inspection no one felt strongly lacking in confidence at time point 2 and by the last time point, 15% of VR group participants felt strongly confident in the procedure. Comparatively, 54% of the control group felt at least mild confidence at time point 2 which increased to 61% by time point 3. Control groups showed a larger proportion (38%) of participants having a strong lack of confidence at time point 2 which was still found at time point 3 and only 8% of control participants had a strong feeling of confidence by time point 3. 

**Figure 2:**
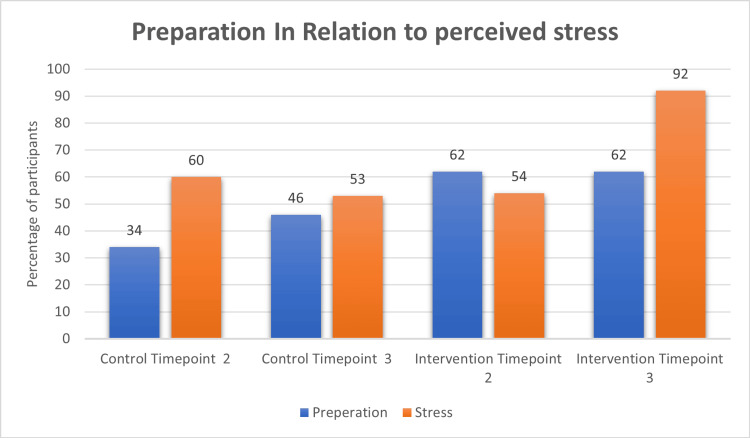
Comparison of association between self-perceived preparation and self-perceived stress between control and intervention group

When looking at stress levels (Figure 2) of the control group, 60% of participants stated they would feel stressed about inserting a TFNA at time point 2 which decreased to 53% by time point 3.

In terms of the extremes of emotion, 27% moderately felt they would not be stressed at time point 2, which by time point 3 was added onto by 7% who strongly felt they would not be stressed. Meanwhile only 54% of intervention group participants felt they would be stressed at time point 2, but this increased by a large amount to 92% at time point 3.

When assessing participant anxiety levels on beginning the study, the central tendency was a feeling of being moderately calm with 82% feeling moderately or very calm. The collated group data also showed a median response of not feeling tense, upset, or worried at all and the calculated modal data supported this.

Median scores indicated participants felt moderately relaxed and content, whilst analysis of pre-study STAI-6 scores showed that there were varied anxiety levels among the cohort (33% low anxiety, 44% moderate anxiety, high anxiety 22%).

By the final time point, the total anxiety levels of the study participants and the low anxiety percentage became 19% whilst the high anxiety percentage increased to 41%, suggesting that overall, for the entire participant group there was a general increase of anxiety from the process.

**Figure 3:**
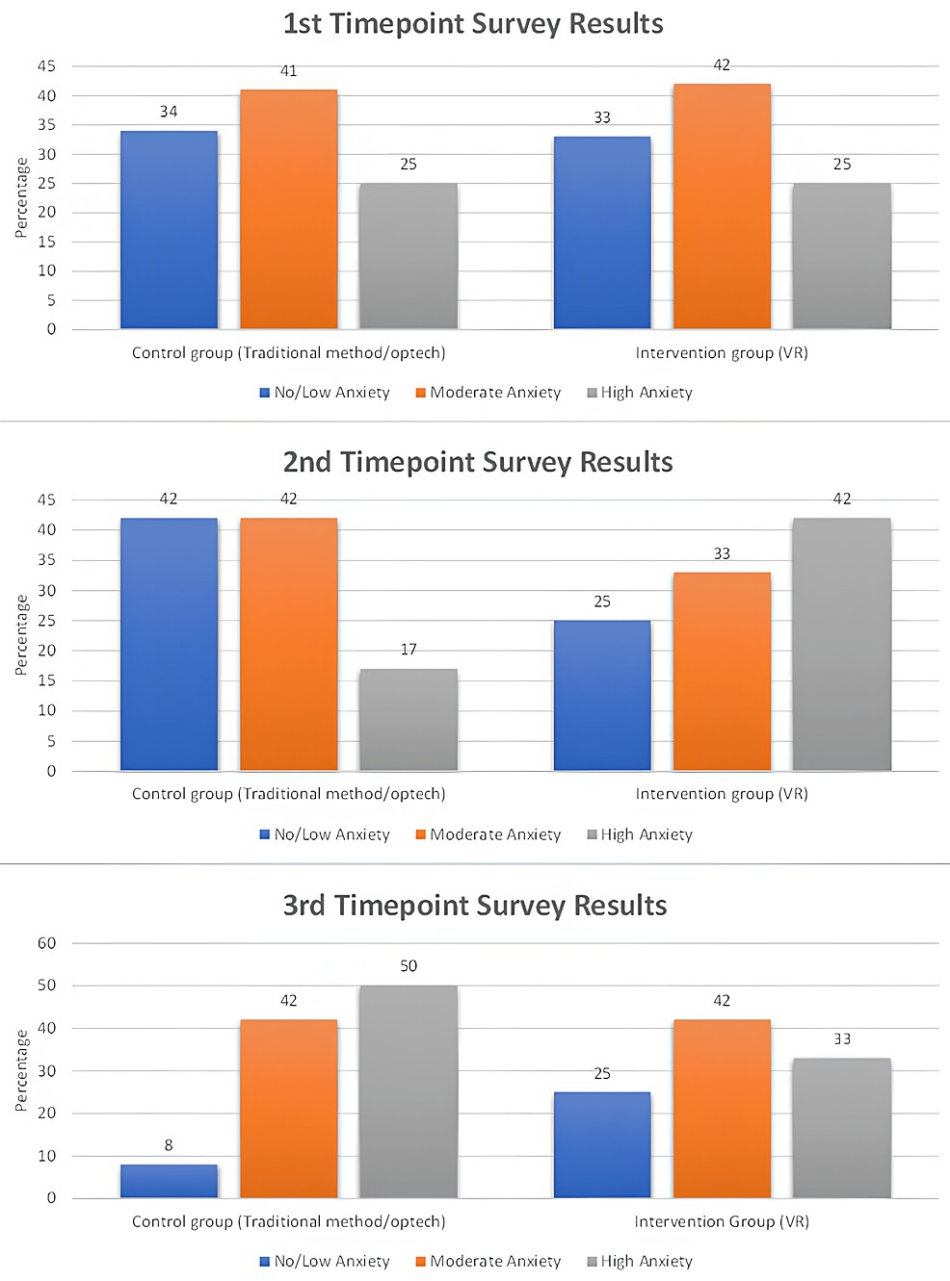
Depiction of STAI-6 result distributions between control and intervention groups over three time points. STAI-6: State-Trait Anxiety Inventory

When then focussing on the anxiety of participants in terms of the separate groups, the changes in anxiety followed the same general trend of increasing but there were differing distributions between them as seen in Figure 3.

Physiological Analysis

Our findings suggest that, while there were no differences in practical skill indicators, there may be positively correlating psychological effects among those who used VR training compared to users of traditional techniques. Hence, we analysed the positive and negative indicators for STAI-6 across all three time points: before completing any study activity (Questionnaire 1 - Q1), after completing the training activity using traditional methods or VR (Questionnaire 2 - Q2), and after completing the bone-model insertion activity (Questionnaire 3 - Q3) using an analysis of variance (ANOVA) test. All scores are displayed as mean (SD).

The traditional methods positive STAI-6 indicators demonstrated an overall increase in positive indicators (Q1 = 2.67 (1.0), Q2 = 2.56 (0.89), Q3 = 2.80 (1.10), p = 0.43). On the other hand, the VR STAI-6 scores showed Q1 = 2.83 (0.73), Q2 = 3.02 (0.61), Q3 = 2.97 (0.73), p = 0.44, with a better outcome at the final time point.

As for the negative indicators, the group using traditional methods showed an overall decline (Q1 = 1.33 (0.60), Q2 = 1.42 (0.62), Q3 = 1.26 (0.68), p = 0.41), which was better than the outcome measured at the final time point for the intervention group (Q1 = 1.36 (0.72), Q2 = 1.19 (0.57), Q3 = 1.30 (0.68), p = 0.48).

## Discussion

This study is the first to study VR in orthopaedic trauma. It uses a mixed-methods approach in assessing both psychological and practical effects of VR simulation in comparison to current UK training techniques.

There has been previous research into the comparison of VR to other methods of surgical training [37-40] in various fields. Our findings suggest a high acceptability of VR, with face and content validity of the simulator plus a united desire for VR within formal training amongst our participant population. The demographics of this population closely resemble the UK orthopaedics trainee population, for instance in sex (21% of general orthopaedic population are females compared to 19% in this study [41]), which further supports generalisability of these attitudes. The same company (Stryker) and implant were used for VR and traditional training resources to minimise differences in instruction and allow for consistency between groups. Our study observed more indicators of positive performance by the VR simulation-trained group, however this was not statistically significant.

Other studies in the same area focus primarily on the surgical performance of their participants using objective metrics such as time taken [31,42]. This study explores more wholesome metrics such as stress and anxiety. Psychological factors within surgeon activity are being increasingly explored as it has been shown that surgeons perceive that surgical performance anxiety can have negative effects on surgical performance [42] as well as adversely affecting surgeon well-being. The Royal College of Surgeons has also highlighted the need for more research in preoperative anxiety [42,43]; this is along with investigating the impact of psychological factors on surgical outcomes as it is already known that interoperative stressors can impact surgical performance [43]. In this growing topic area, our study shows that VR may also be an advantageous way to tackle these issues within training.

It has been noted that surgeons tend to be kinaesthetic learners [44] which was supported by the preferences voiced by our participants. It has also been seen that learning environments can directly influence the ability of a person to learn [45] and optimising this decreases stress, as set up in our study, which facilitates effective learning. To enable the optimum learning environment, it is imperative for the simulated environment to be as close to as true a representation of the task at hand. This would include as high a face validity as possible [46] as well as appropriate variables contained within the simulation and user compatibility with the target audience [47]. This study has shown a high acceptability of this simulator by users, suggesting high face validity as well as user compatibility.

The effects on confidence seen in the studied groups, particularly the positive effect within our intervention group, may be due to a culmination of factors. A randomised control trial [48] showed that mental imagery can be applied to learning tasks by stimulation of neuroplastic motor pathways, and when used in a surgical context, may positively impact user self-confidence. VR has also been shown to activate neuroplastic pathways in multiple areas of the brain dependent on the simulation used [6,49] including the pre-frontal and motor cortices, possibly sharing a similar mechanism with mental imagery towards improving self-confidence. This may prove beneficial as self-confidence has been shown to correlate positively with performance and operative success in multiple specialties [50]. Interestingly, despite the confidence levels seen within our control group decreasing through the process, they felt less stressed about completing the task. This initially may be surprising until paired with how prepared participants felt for the procedure, as it was seen that the control group felt much less prepared than the intervention group at each time point. This may thus indicate a lack of self-perceived realistic awareness within the control group, potentially due to reduced familiarity, in a similar fashion to junior trainees when compared to seniors in other studies [51], as despite feeling unprepared for the procedure they also felt they would not be stressed attempting it.

These results also validate this VR simulator with regard to content validity. The vast majority believed that most components included within the VR system were accurate, assisted with their understanding and helped them feel more prepared. The fact that the intervention group found VR highly enjoyable may also be reflected in their STAI-6 outcomes showing generally lower and more stable anxiety levels. The differences between the groups may thus also be due to an inaccurate perception of the procedure’s difficulty and as such an overconfidence in the control group’s skill. This would align with Akhtar et al.'s findings of reduced user awareness seen in the practical skills of surgical novices in hip surgery [52]. 

The overwhelming opinion from both groups was towards making virtual simulation compulsory in training. The fact that virtual reality has been adopted in other countries adds further weight to its introduction in UK surgical training [12,46,52]. However, further evaluation of a proposed training scheme, including transfer validity to real-life surgery, is required.

Limitations

This study is limited by the small sample size, due to the limited number of course attendees and equipment availability. This may have inhibited the ability for statistical significance to be exhibited. Whilst a training time of 20 minutes was similarly used in other studies [14,52] it could be considered that this amount of time was not universally sufficient to adequately upskill participants. 

Future work

As this study shows good face-content validity and acceptability of VR simulation, it can form the basis for further evaluation of the simulator. This study explored two groups; however, implementation of simulation may incorporate access to both VR and traditional methods. As such, an appropriately powered three-armed study may provide further information about the potential of VR simulation. Further assessments of the construct validity, the training effect of the simulator and ultimately the transfer validity of surgical skills to the operating theatre are also required. Exploration using qualitative methods may provide a richer understanding of the perception, benefits, and potential barriers of simulation training.

## Conclusions

The utilization of VR simulations creates a non-threatening and highly immersive learning environment, one that is both realistic and readily embraced by surgical trainees. In our study, the integration of the DePuy Synthes Oculus Rift S significantly bolstered trainee confidence and simultaneously mitigated trainee anxiety levels. This compelling dual-effect underscores the potential for VR to be seamlessly integrated into the surgical training curriculum. Furthermore, this innovative approach garnered unanimous support and enthusiasm within our specific study population, affirming its feasibility and desirability as an integral component of surgical education. In light of these findings, VR's incorporation into surgical training not only seems warranted but also promises to be a universally embraced and beneficial addition.
